# Headache in relapse and remission phases of multiple sclerosis: A case-control study

**Published:** 2016-01-05

**Authors:** Mansoureh Togha, Nahid Abbasi Khoshsirat, Abdorreza Naser Moghadasi, Faezeh Mousavinia, Mohammad Mozafari, Mohamadreza Neishaboury, Seyed Mahmood Mousavi

**Affiliations:** 1 Headache Research Center, Iranian Center of Neurological Research, Neuroscience Institute AND Department of Neurology, Sina Hospital, Tehran University of Medical Sciences, Tehran, Iran; 2 Headache Research Center, Iranian Center of Neurological Research, Neuroscience Institute, Tehran University of Medical Sciences, Tehran, Iran; 3 Department of Neurology, Sina Hospital, Tehran University of Medical Sciences, Tehran, Iran

**Keywords:** Multiple Sclerosis, Headache, Relapse, Remission, Case-Control Studies

## Abstract

**Background:** Headaches are one of the most frequent reasons for pain in multiple sclerosis (MS) individuals. Characterization of headaches and delineating possible relationships with MS-related determinants can ultimately circumvent headaches.

**Methods:** In a prospective case-control study, 65 Iranian relapsing-remitting MS (RRMS) patients and 65 healthy controls were recruited during patients’ admission for attack-period treatment and asked about characteristics and co-symptoms of headaches they experienced in the preceding week and usage of disease modifying drugs (DMDs) and types of MS attacks were also inquired. The same questions were asked from the same patients 3 months later in a follow-up visit.

**Results:** A total of 57 patients and 57 controls were included in the final analyses. In total, 26 (45.6%) patients in relapse, 18 (27.7%) controls, and 22 (38.6%) patients in remission reported headaches and only significant difference existed between relapse patients and controls (P = 0.036). In headache prevalence was higher in patients in relapse phase having MS < 3 years compared to relapse patients with more than 3 years of MS (68 vs. 28.1%; P = 0.004). Other variables of interest did not differ among the three groups.

**Conclusion:** The RRMS patients in relapse phase suffer from headaches more than healthy people do.

## Introduction

Amid the plethora of multiple sclerosis (MS) symptoms,^1^^,^^2^ pain is vividly recalled by patients and apparently accompanies them throughout the entire disease period.^3^^,^^4^ The latest systematic review considering prevalence of pain in adult MS subjects declared an overall prevalence of 63.0%; the most common instigator of pain in this meta-analysis was headache, with a prevalence of about 43.0%.^3^ The very first documentation of headache occurrence in MS patients was revealed over 60 years ago.^5^ Following this pioneer initiative, the association of MS and primary headaches has been commonly reviewed, and headache prevalence rates ranging from 4.0 to 76.3% have been acknowledged in MS patients.^2^^,^^6^^-^^19^ Migraine without aura and tension-type headache (TTH) are the top two prevalent primary headaches seen in MS populations.^10^^,^^20^ In addition, stabbing headaches are more commonly seen in relapses of relapsing-remitting MS (RRMS) subjects and migraine and TTH comprise most of the headaches experienced by RRMS individuals during remissions.^1^^,^^21^ Of note, other types and manifestations of primary headaches such as cluster headache, migraine-like headache, cluster-like headache, and status migrainosus and also secondary headaches have been observed in MS patients.^6^^,^^7^^,^^10^^,^^22^^-^^25^

The sprouting of headaches during interferon-beta (INF-) therapy has been cited by MS subjects as a major rationale for terminating INF- use^26^ and as RRMS patients are frequently given INF- for their neurologic symptoms,^11^ delineating the relationship between headache deterioration or establishment in RRMS and INF- is important.

The emotional burden of headaches in MS patients is significant and those suffering from headaches throughout their disease period are likely entitled to a plunge in quality of life.^11^^,^^27^^,^^28^ In particular, MS patients simultaneously affected by migraine will experience more symptoms of MS and eventually have poorer quality of life.^29^^,^^30^ Furthermore, primary stabbing headache (PSH) has been proposed as a premonitory sign of acute demyelinating fallout in RRMS.^1^^,^^21^^,^^27^ Consequently, rapid recognition and management of headaches in MS patients can potentially be of utmost importance. Studies evaluating headaches happening during relapses and remissions of RRMS and depicting the characteristics of these headaches and their possible relationship with different MS attack variants and factors such as depression and disease modifying drugs (DMDs) at the same time are scarce. Thus, in this current effort, we attempted to assess the prevalence of headaches during relapses and remissions of RRMS patients and define the attributes of these headaches and denote the possible interrelations between headaches and therapeutic determinants, co-symptoms such as depression and types of MS exacerbations.

## Materials and Methods

A prospective study was initiated to assess characteristics of headaches in patients with RRMS visited in Sina Hospital (affiliated to Tehran University of Medical Sciences) in Tehran, Iran, between 2013 and 2015. Eligible patients for enrollment as case group included those with pre-existing MS experiencing an acute relapse of disease. The cases chosen were not randomly selected and were, in fact, available cases consecutively picked from the RRMS patients checking in for acute relapse treatment during the study period. Diagnosis of MS has been made according to the latest version of McDonald criteria.^31^ Furthermore, diagnosis of types of headaches seen in MS patients and controls was in accordance with third edition (beta version) of the International Classification of Headache Disorders.^32^ The control group was comprised of healthy individuals that were chosen consecutively and not randomly from people accompanying RRMS patients in the Neurology Clinic of Sina Hospital. These controls had no previous history of neurologic disorders such as MS, seizure and severe head trauma and were not affected by other chronic diseases such as hypertension, renal failure, etc. Individuals with these attributes were then asked if they suffer from headaches and if the answer was “yes,” characteristics of their headaches would be entered in assessment forms and recorded. If the answer was “no,” then only demographic data pertinent to them would be documented. Frequency matching was performed to have a control group similar to case group with respect to gender distribution and age (± 5 years). The study protocol was approved by the Ethics Committee at Tehran University of Medical Sciences and written informed consent was obtained from all study participants prior to enrollment.

Demographic information, current treatment protocol and DMDs used during the past 6 months were recorded. If patients were experiencing headache(s) during the past week, a predesigned structured questionnaire was used to record the features of the headache(s). The types of headaches documented were migraine, TTH or secondary headache. The severity of headache was assessed by a 10-point visual analogue scale (VAS), in which 1 indicates the lowest severity and 10 is considered as very severe headache. The VAS score was further categorized as follows: scores of 1, 2 and 3 were considered as mild, 4-7 as moderate and severe headache as VAS score of 8, 9 or 10. The quality of pain was recorded as compressing, pulsatile and stabbing. Patients were asked about the location of headache that could be orbital, frontal, occipital, vertex and a combination of mentioned sites. In addition, the presence of concomitant symptoms such as photophobia, phonophobia and nausea and vomiting were checked. Of note, the type of MS attack in relapse phase including blurred vision (optic neuritis), myelitis (spinal lesions), hemiparesis/hemihypoesthesia (hemispheric lesions) and symptoms such as ataxia, gaze palsy, crossed cranial nerve palsy and motor or sensory symptoms (brainstem and cerebellar lesions) was obtained from patients’ files and recorded. Participants in the control group were also asked about any episodes of headache during the past 7 days and its features.

The following completion of the treatment protocol for acute relapse, patients were discharged and scheduled for a follow-up visit. The visit was set up for 3 months later to assess patients in remission phase of MS. Patients were excluded if they experienced any episodes of relapse during this period. The patients were requested to fill in the follow-up sheet questionnaire that included questions about headache characteristics and any drugs consumed for headache prophylaxis or acute attacks. RRMS patients were also inquired about other non-DMD medications they took during remission period. 

SPSS software for Windows (version 20, SPSS Inc., Chicago, IL, USA) was employed to perform the statistical analyses. Continuous variables are presented as mean and standard deviation and categorical variables are shown as percentage. The frequency of headaches was compared between case and control groups using chi-square and Fisher’s exact tests both in baseline and follow-up visits. Within group comparison using McNemar’s test was performed to evaluate any changes in the prevalence and characteristics of headaches in patients with MS during acute relapse and remission of the disease. P < 0.050 was considered as statistically significant in all tests.

## Results

A total of 130 patients were enrolled (65 patients in each group). During the follow-up period, 8 patients in case group experienced another relapse attack and were excluded from the study and a total of 57 patients from case group were included in the final analyses. The median duration of disease was 4 years (interquartile range: 1.5-8.0). Baseline characteristics of study population are presented in table 1.

Among the patients in case group, 26 (45.6%) patients reported having headaches during the 7 days preceding admission for treatment of MS flare-up, of which 25 (96.1%) were females and 1 (3.9%) was a male. This number decreased to 22 (38.6%) patients in follow-up visit (remission). The corresponding figure in control group was 18 (27.7%) and out of the 18 control patients suffering from headaches, 4 (22.2%) were males and 14 (77.8%) were females. The frequency of headaches was significantly higher in patients with RRMS during relapse phase compared with control group (P = 0.036). Considering remission period, there were no differences comparing the mentioned figures between the study groups (P = 0.247). Taking headache-gender relation into account, no significant association was witnessed either in control (P = 0.277), relapse (P = 0.132) or remission group (P = 0.0.87).

Among patients with RRMS who experienced headaches during their relapses, the most common type of headache was migraine (n = 16, 28.1%) followed by TTH (n = 10, 14%). Most patients reported the severity and quality of their headaches as severe (22.8%) and compressing (28.1%), respectively. Fronto-orbital was the most common location of headache, as reported by 10 (17.5%) patients. Finally, 15 (26.3%) patients reported that they were experiencing photophobia and phonophobia concomitantly and 8 (14.1%) patients complained of nausea and/or emesis. In the control group, 12 (18.4%) of the headache sufferers were diagnosed with migraine and 6 (9.2%) of them had TTH. Furthermore, the majority of controls declared their headaches to be moderate and pulsatile (13.8 and 18.4%, respectively). In addition, most headache subjects in control group (n = 5, 7.7%) experienced frontal headaches. At last, 6 (9.2%) of the controls noted accompanying phonophobia and photophobia and 8 (12.3%) controls had nausea and/or vomiting as co-symptoms during their headaches. Comparing the participants in control group with headaches and RRMS patients in relapse phase with headaches, none of the aforementioned headache characteristics differed between the two groups significantly and this finding also held true for type of headaches and headache co-symptoms (all corresponding P values> 0.050).

**Table 1:** Baseline characteristics of study population

**Characteristics**	**Case (n = 57)**	**Control (n = 65)**
Age (mean ± SD)	31.84 ± 9.32	36.37 ± 11.34
Gender [n (%)]		
Male	8 (14.0)	10 (15.4)
Female	49 (86.0)	55 (84.6)
Educational level [n (%)]		
College graduate	13 (22.8)	12 (18.4)
High school diploma	22 (38.6)	30 (46.2)
Less than high school	9 (15.8)	17 (26.2)
Missing	13 (22.8)	6 (9.2)
Marital status [n (%)]		
Married	30 (52.6)	41 (63.1)
Single	15 (26.3)	16 (24.6)
Missing	12 (21.1)	8 (12.3)

SD: Standard deviation

**Figure 1:**
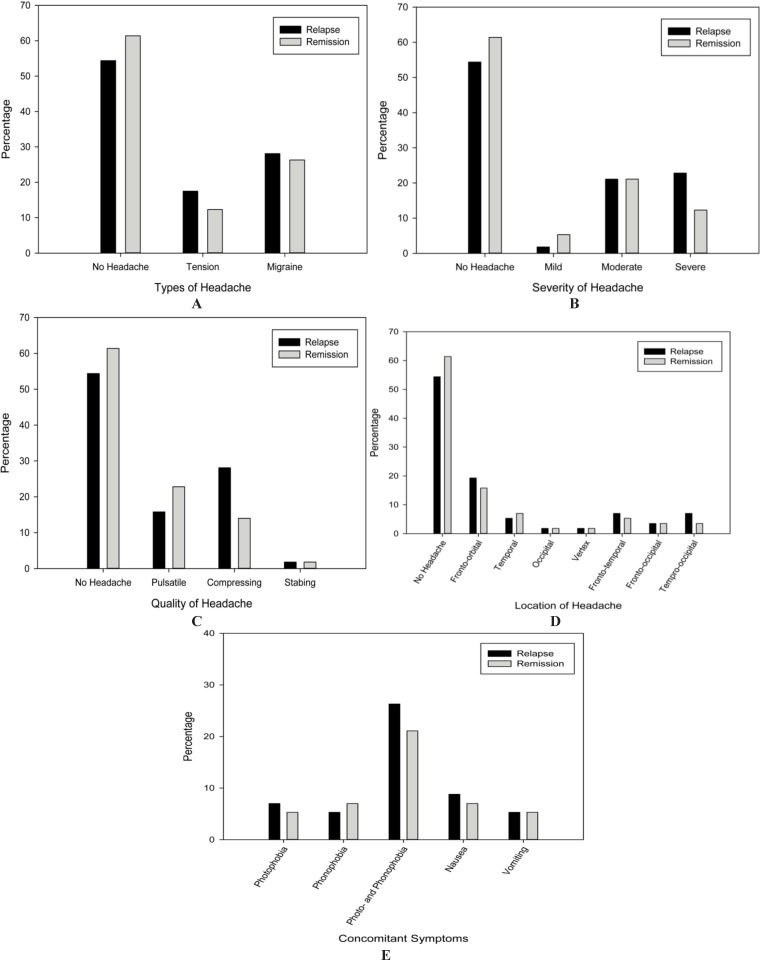
Features of headache attacks during relapse and remission phases among patients with relapsing-remitting multiple sclerosis, (A) Types of headache, (B) severity of headache, (C) quality of headache, (D) location of headache, (E) concomitant symptoms

About 3 months after the flare-up of the disease and during the remission period, the most frequent type of headache was still migraine type (26.3%), but the severity of the headaches decreased and 12 (21.1%) patients reported they were experiencing moderate headaches during the remission follow-up period. No major change was observed in the common location and quality of headaches during these 3 months. Detailed information regarding the features of headache attacks during relapse and remission phases in case group are presented in figure 1 (A-E). 

Comparing features of headaches between the two phases via McNemar’s test, no significant differences regarding type (P = 0.379), severity (P = 0.528), quality (P = 0.092), or location of headaches (P = 0.506) and presence of concomitant symptoms including photophobia and phonophobia (P = 0.669) or nausea and/or vomiting (P = 0.905) were detected. To be noted, none of the controls or RRMS patients who had headaches used preventive medication for headaches, but two controls (3.1%) and three MS patients (5.7%) encountered medication overuse headache (MOH). Moreover, five headache-positive RRMS patients (8.7%) and two headache-negative MS cases (3.5%) used anti-epileptics and anti-depressants (tricyclic anti-depressants) for pain and psychiatric purposes during remission phase, respectively.

Patients were divided into two groups based on the duration of disease; 32 (56.1%) patients were diagnosed with MS more than 3 years ago and 25 (43.9%) subjects were diagnosed during the past 3 years. Seventeen patients (68.0%) in the latter group reported headaches during flare-up of disease, which was significantly higher than the frequency of headaches among patients with disease-duration of more than 3 years (n = 9, 28.1%) (P = 0.004). Such association was not observed for the prevalence of headaches during remission phase (52.0 vs. 28.1%, P = 0.066).

Of the 26 RRMS patients who suffered from headaches during their relapses, 8 (14.1%) of them went through an optic neuritis flare-up, 10 (17.5%) subjects experienced hemiparesis/hemihypoesthesia, 4 (7.1%) individuals faced brainstem or cerebellar symptoms during their flare-up and 4 (7.1%) patients suffered a myelitis attack. For their no-headache MS counterparts, the following numbers were seen: nine patients (15.8%) with blurred vision at attack, 14 (24.6%) individuals with hemispheric lesions at relapse, 7 (12.3%) subjects with cerebellar and brainstem lesions at flare-up and 9 (15.8%) patients with myelitis at exacerbation of MS. Ultimately, there was no significant correlation between a specific type of MS attack and headache prevalence (P = 0.273).

From the DMD perspective, four RRMS patients stated that their headaches got worse after INF- treatment [1 used Ziferon (INF- 1b), 2 treated with ReciGen (INF- 1a) and 1 consumed CinnoVex (INF- 1a)] (Iranian version of Betaferon, Rebif and Avonex). On the other hand, none of the MS patients using fingolimod or glatiramer acetate (GA) noticed headache exacerbation after DMD therapy.

Another finding that should be mentioned is that in one of the RRMS patients, exacerbation of a pre-existing migraine headache was the possible first presentation of MS, as it forced the patient to seek medical care and led to the diagnosis of MS in that patient.

## Discussion

Nowadays, the notion that headaches of different types are associated with MS has gained acceptance and this specifically goes for migraine.^21^ Nevertheless, the properties of this association still remain vague. Scientists have delved into the enigmatic pathophysiological and epidemiological relationship between headaches and MS and most of these efforts have been made in the area of migraine and MS. About half a century ago, Watkins et al. carried out a case-control study in which they demonstrated that MS subjects are 2 times more likely to have positive family history of migraine compared to healthy controls.^16^ Zorzon et al.^33^ confirmed this finding as they too declared that people with migraine running in their family have a higher chance of developing MS. 

In the early 90s, when three MS individuals experienced migraine during MS flare-up, serotonin was incriminated for bridging the gap between MS and migraine; serotonin was implicated earlier before in migraine pathology and as MS patients were already “out of” serotonin, serotonin perturbations could have possibly played a role in MS attacks.^34^ Simple co-morbidity of headaches like migraine and MS is another elucidation in the headache-MS path.^19^^,^^30^^,^^33^ Another piece of evidence for common ground between MS and headaches is the drop in circulating T8 lymphocytes in both settings.^35^^,^^36^ Pregnancy is another part of the thread between MS and migraine, as both pregnant migraineurs and pregnant MS patients enjoy a less debilitating course of disease during pregnancy.^37^ In the mid-2000s, neurologists laid out an explanation for migraine-MS interrelationship; they suggested that migraine, especially migraine with aura, switches on particular matrix metalloproteinases that in turn destruct the blood-brain barrier and render myelin autoantigens vulnerable to autoreactive T-cells present in the bloodstream and this process can ultimately culminate in MS.^38^^,^^39^ Furthermore, migraine episodes and MS attacks apparently share common cytokine profiles.^19^^,^^40^ Common genetic and environmental determinants present in MS, migraine and TTH are also on the agenda of headache-MS association.^8^

The prevalence rate of headaches in relapse and remission subsets of our study was 45.6% and 38.6%, respectively, which is in accord with the last systematic review on headaches in MS patients, that announced a crude headache prevalence rate of 42.5% in MS patients.^3^ However, two distinct studies in the past 7 years have manifested high headache prevalence numbers in RRMS populations, i.e., 74.7% (83 RRMS subjects) and 76.3% (118 RRMS patients), in order.^14^^,^^15^ In this current study, we showed that our RRMS patients confronted headaches in their relapse phases significantly more frequent than the controls did. On the contrary, RRMS patients failed to retain this significance in remission period, although they did encounter headaches more often than controls. Furthermore, headache prevalence rates in relapse and remission time periods were comparable in our effort. Ergun et al.^1^ conducted a study in which they assessed headaches in two separate sets of relapse phase and remission phase-RRMS patients and they declared that a significantly higher proportion of RRMS patients in remission period complained of headaches compared to their relapse phase-RRMS counterparts (73.5 vs. 38.9%, respectively). 

The difference witnessed between the prevalence rates of their study and ours can be explained by the fact that the number of subjects in relapse and remission in their study were approximately a half and a third of ours, respectively, and this subsequently lowers the power of their study. Furthermore, their relapse and remission patients were two distinct groups of people, but our relapse phase-RRMS patients were the same ones assessed in remission period. Therefore, intervening factors related to different sets of patients could have skewed the results of Ergun et al.’s study.^1^ In addition, no healthy controls took part in their study.

Taking types of headaches into account, no significant difference was observed comparing those in remission and relapse and controls; more than 60.0% (28.1% of all RRMS patients in relapse) of headaches in our relapse period were of migraine type and the rest were TTHs and the percentage of migraineurs in remission and control subsets was 68.0 and 66.6%, respectively. Conversely, PSHs (27.8%) constituted most headaches seen in relapse patients of Ergun et al.’s study,^1^ with migraine and MOH counting for 11.1 and 5.9% of patients in relapse, in order. 

However, the distribution of types of headaches in remission was similar to ours (i.e., migraine 41.2% and TTH 20.6% of patients). Two distinct RRMS cohorts reported prevalence of migraine to be 49.8 and 34.1%, in order, and 27.17% of subjects in the latter group were diagnosed with TTH.^41^^,^^42^ In a questionnaire-based study carried out in 2013, 46% of 673 MS patients announced that they were battling with migraine and migraine headaches significantly accompanied RRMS patients.^4^ Villani et al.^14^ and D’Amico et al.^6^ have also demonstrated a significant association between RRMS and migraine headache. 

In a meta-analysis performed by Pakpoor et al.,^28^ MS patients displayed an over two-fold increment in likelihood of acquiring migraine headaches compared to controls. In addition, a cross-sectional study showed that the chance MS patients have for confronting co-morbid migraine is trice than controls.^30^ Contradictory to these studies, two different case-control studies^13^^,^^19^ carried out stated in 2007 and 2009 non-significant difference between MS cases and controls regarding headache type distribution. Katsiari et al.^43^ also noticed no significant correlation between exacerbations of MS and other autoimmune diseases and different categories of headaches. 

Considering severity and quality of headaches, most of our RRMS patients in relapse had severe and compressing headaches, which were alleviated by remission and turned into moderate headaches in that period. Nevertheless, no significant difference with regard to these factors existed when controls and patients in remission and relapse were compared and this insignificance was replicated when assessing photophobia/phonophobia, nausea and/or vomiting and location of headaches in the groups. With the aid of a questionnaire, Tabby et al.^27^ indicated that headache-related pain in their 72 MS patients was throbbing, stabbing or sharp most of the times and bilateral headaches were more common than unilateral ones. In Moisset et al.'s survey^4^ most MS patients notified the researchers that their headaches were moderately painful.

In the study of Moisset et al.^4^ patients who were diagnosed with MS < 15 years before the time of study had a higher probability of having migraine. In our current effort, we also showed that RRMS patients in relapse phase with shorter disease-duration (< 3 years) were more prone to headaches than those coping with RRMS for more than 3 years. Of note, we did not detect this significant relationship in subjects in remission. One explanation for this finding could be that MS patients are less neurologically-disabled in their first years and therefore, pay more attention to headaches compared to those suffering from more neurological-related disabilities that prevent them from correctly detecting the presence of headaches. From another point of view, the neuro-inflammation played out in the first couple of years of RRMS could possibly explain the association between RRMS and headache occurrence in these years.

Our results indicated that most of the RRMS patients, with or without headaches, experienced optic neuritis attacks in relapse. However, no significant association between type of attack (and subsequently site of involvement by MS lesions) and headache prevalence was found. In a cohort of 127 Japanese MS patients, no significant correlation between brainstem-spinal, optico-spinal, and conventional MS clinical variants and headache prevalence was detected.^7^ In the aforementioned Ergun et al. study,^1^ total headache occurrence was significantly linked to periventricular lesions, TTHs happening in remission were more frequently associated with lesions involving the spinal cord and overall headache incidence in relapse was significantly intertwined with brainstem lesions. In a retrospective research, Gee et al.^44^ declared that MS patients with lesions found in the periaqueductal grey (PAG)/midbrain area are more likely to suffer from migraine-like headaches and TTHs. Several case-reports have revealed a possible association between brainstem and PAG MS plaques and migraine incidence^45^^,^^46^ and several other have demonstrated a link between cluster headache occurrence and MS lesions infiltrating trigeminal nerve entry zone in the brainstem.^22^^,^^47^

More than a decade ago, speculations were made regarding the role of DMDs in exacerbating headaches in MS patients^12^ and current scientific evidence points out a significant positive correlation between emergence of new headaches or worsening of old headaches in MS patients and INF- treatment.^42^^,^^48^^-^^50^ In a recent report, novel fingolimod usage in MS patients has been linked to the happening of new headaches.^51^ Natalizumab was another DMD investigated in this regard and Villani et al.^48^ announced that it does not make comorbid migraines worse in those suffering from MS. In addition, researchers have claimed that GA does not significantly increase the frequency or severity of headaches in MS subjects who already have headaches.^12^^,^^52^ An important limitation that we faced, in our study, was that patient information regarding use of DMDs was incomplete and many of them did not use these medications on a regular basis and although four patients using INF- mentioned exacerbation of headaches after this kind of therapy and not a single patient experienced aggravation of their headache post-GA and fingolimod consumption, proper statistical evaluation was not possible.

Headache can be the presenting symptom of MS, as we have also showed in this study. Therefore, prompt intervention at headache onset in MS patients with contrast-enhanced magnetic resonance imaging could reveal novel plaques and if so subsequent pulse of methylprednisolone can prevent an ensuing MS relapse.

## Conclusion

We suggest that RRMS patients in relapse phase suffer from headaches more than the general population does and those who complain of headaches in relapse phase have probably been diagnosed with RRMS in the preceding 3 years.

## Notes:

**How to cite this article:** Togha M, Abbasi-Khoshsirat N, Naser-Moghadasi A, Mousavinia F, Mozafari M, Neishaboury M, et al. Headache in relapse and remission phases of multiple sclerosis: A case-control study. Iran J Neurol 2016; 15(1): 1-8**.**

## References

[1] Ergun U, Ozer G, Sekercan S, Artan E, Kudiaki C, Ucler S (2009). Headaches in the different phases of relapsing-remitting multiple sclerosis: a tendency for stabbing headaches during relapses. Neurologist.

[2] Nicoletti A, Patti F, Lo Fermo S, Liberto A, Castiglione A, Laisa P (2008). Headache and multiple sclerosis: a population-based case-control study in Catania, Sicily. Cephalalgia.

[3] Foley PL, Vesterinen HM, Laird BJ, Sena ES, Colvin LA, Chandran S (2013). Prevalence and natural history of pain in adults with multiple sclerosis: systematic review and meta-analysis. Pain.

[4] Moisset X, Ouchchane L, Guy N, Bayle DJ, Dallel R, Clavelou P (2013). Migraine headaches and pain with neuropathic characteristics: comorbid conditions in patients with multiple sclerosis. Pain.

[5] Mcalpine D, Compston N (1952). Some aspects of the natural history of disseminated sclerosis. Q J Med.

[6] D'Amico D, La ML, Rigamonti A, Usai S, Mascoli N, Milanese C (2004). Prevalence of primary headaches in people with multiple sclerosis. Cephalalgia.

[7] Doi H, Matsushita T, Isobe N, Ishizu T, Ohyagi Y, Kira J (2009). Frequency of chronic headaches in Japanese patients with multiple sclerosis: with special reference to opticospinal and common forms of multiple sclerosis. Headache.

[8] Kister I, Caminero AB, Herbert J, Lipton RB (2010). Tension-type headache and migraine in multiple sclerosis. Curr Pain Headache Rep.

[9] Martinelli BF, Colombo B, Annovazzi P, Martinelli V, Bernasconi L, Solaro C (2008). Lifetime and actual prevalence of pain and headache in multiple sclerosis. Mult Scler.

[10] Mijajlovic MD, Aleksic V, Covickovic Šternic NM (2014). Cluster headache as a first manifestation of multiple sclerosis: case report and literature review. Neuropsychiatr Dis Treat.

[11] Mohrke J, Kropp P, Zettl UK (2013). Headaches in multiple sclerosis patients might imply an inflammatorial process. PLoS One.

[12] Pollmann W, Erasmus LP, Feneberg W, Then BF, Straube A (2002). Interferon beta but not glatiramer acetate therapy aggravates headaches in MS. Neurology.

[13] Putzki N, Pfriem A, Limmroth V, Yaldizli O, Tettenborn B, Diener HC (2009). Prevalence of migraine, tension-type headache and trigeminal neuralgia in multiple sclerosis. Eur J Neurol.

[14] Villani V, Prosperini L, Ciuffoli A, Pizzolato R, Salvetti M, Pozzilli C (2008). Primary headache and multiple sclerosis: preliminary results of a prospective study. Neurological Sciences.

[15] Villani V, Prosperini L, Pozzilli C, Salvetti M, Ciuffoli A (2011). The use of ID migraine™ questionnaire in patients with multiple sclerosis. Neurological Sciences.

[16] Watkins SM, Espir M (1969). Migraine and multiple sclerosis. J Neurol Neurosurg Psychiatry.

[17] Yetimalar Y, Secil Y, Inceoglu AK, Eren S, Basoglu M (2008). Unusual primary manifestations of multiple sclerosis. N Z Med J.

[18] Freedman MS, Gray TA (1989). Vascular headache: a presenting symptom of multiple sclerosis. Can J Neurol Sci.

[19] Vacca G, Marano E, Brescia Morra V, Lanzillo R, Vito M, Parente E (2007). Multiple sclerosis and headache co-morbidity. A case-control study. Neurological Sciences.

[20] La ML, Prone V (2015). Headache in multiple sclerosis and autoimmune disorders. Neurol Sci.

[21] Klein M, Woehrl B, Zeller G, Straube A (2013). Stabbing headache as a sign of relapses in multiple sclerosis. Headache.

[22] Leandri M, Cruccu G, Gottlieb A (1999). Cluster headache-like pain in multiple sclerosis. Cephalalgia.

[23] Alroughani R, Ahmed SF, Khan R, Al-Hashel J (2015). Status migrainosus as an initial presentation of multiple sclerosis. Springerplus.

[24] Rampello L, Malaguarnera M, Rampello L, Nicoletti G, Battaglia G (2012). Stabbing headache in patients with autoimmune disorders. Clin Neurol Neurosurg.

[25] Mariotti P, Nociti V, Cianfoni A, Stefanini C, de Rose P, Martinelli D (2010). Migraine-like headache and status migrainosus as attacks of multiple sclerosis in a child. Pediatrics.

[26] Tremlett HL, Oger J (2003). Interrupted therapy: stopping and switching of the beta-interferons prescribed for MS. Neurology.

[27] Tabby D, Majeed MH, Youngman B, Wilcox J (2013). Headache in multiple sclerosis: features and implications for disease management. Int J MS Care.

[28] Pakpoor J, Handel AE, Giovannoni G, Dobson R, Ramagopalan SV (2012). Meta-analysis of the relationship between multiple sclerosis and migraine. PLoS One.

[29] Villani V, Prosperini L, Pozzilli C, Salvetti M, Sette G (2011). Quality of life of multiple sclerosis patients with comorbid migraine. Neurological Sciences.

[30] Kister I, Caminero AB, Monteith TS, Soliman A, Bacon TE, Bacon JH (2010). Migraine is comorbid with multiple sclerosis and associated with a more symptomatic MS course. J Headache Pain.

[31] Polman CH, Reingold SC, Banwell B, Clanet M, Cohen JA, Filippi M (2011). Diagnostic criteria for multiple sclerosis: 2010 revisions to the McDonald criteria. Ann Neurol.

[32] Headache Classification Committee of the International Headache Society (IHS) (2013). The International Classification of Headache Disorders, 3rd edition (beta version). Cephalalgia.

[33] Zorzon M, Zivadinov R, Nasuelli D, Dolfini P, Bosco A, Bratina A (2003). Risk factors of multiple sclerosis: a case-control study. Neurological Sciences.

[34] Sandyk R, Awerbuch GI (1994). The co-occurrence of multiple sclerosis and migraine headache: the serotoninergic link. Int J Neurosci.

[35] Leone M, Biffi M, Leoni F, Bussone G (1994). Leukocyte subsets and cortisol serum levels in patients with migraine without aura and chronic tension-type headache. Cephalalgia.

[36] Reder AT, Antel JP, Oger JJ, McFarland TA, Rosenkoetter M, Arnason BG (1984). Low T8 antigen density on lymphocytes in active multiple sclerosis. Ann Neurol.

[37] Pearce CF, Hansen WF (2012). Headache and neurological disease in pregnancy. Clin Obstet Gynecol.

[38] Kister I, Munger KL, Herbert J, Ascherio A (2012). Increased risk of multiple sclerosis among women with migraine in the Nurses' Health Study II. Mult Scler.

[39] Gursoy-Ozdemir Y, Qiu J, Matsuoka N, Bolay H, Bermpohl D, Jin H (2004). Cortical spreading depression activates and upregulates MMP-9. J Clin Invest.

[40] Perini F, D'Andrea G, Galloni E, Pignatelli F, Billo G, Alba S (2005). Plasma cytokine levels in migraineurs and controls. Headache.

[41] Villani V, de Giglio L, Sette G, Pozzilli C, Salvetti M, Prosperini L (2012). Determinants of the severity of comorbid migraine in multiple sclerosis. Neurol Sci.

[42] Patti F, Nicoletti A, Pappalardo A, Castiglione A, Lo FS, Messina S (2012). Frequency and severity of headache is worsened by Interferon-beta therapy in patients with multiple sclerosis. Acta Neurol Scand.

[43] Katsiari CG, Vikelis M, Paraskevopoulou ES, Sfikakis PP, Mitsikostas DD (2011). Headache in systemic lupus erythematosus vs multiple sclerosis: a prospective comparative study. Headache.

[44] Gee JR, Chang J, Dublin AB, Vijayan N (2005). The association of brainstem lesions with migraine-like headache: an imaging study of multiple sclerosis. Headache.

[45] Fragoso YD, Brooks JB (2007). Two cases of lesions in brainstem in multiple sclerosis and refractory migraine. Headache.

[46] Haas DC, Kent PF, Friedman DI (1993). Headache caused by a single lesion of multiple sclerosis in the periaqueductal gray area. Headache.

[47] Gentile S, Ferrero M, Vaula G, Rainero I, Pinessi L (2007). Cluster headache attacks and multiple sclerosis. J Headache Pain.

[48] Villani V, Prosperini L, de Giglio L, Pozzilli C, Salvetti M, Sette G (2012). The impact of interferon beta and natalizumab on comorbid migraine in multiple sclerosis. Headache.

[49] La ML, D'Amico D, Rigamonti A, Mascoli N, Bussone G, Milanese C (2006). Interferon treatment may trigger primary headaches in multiple sclerosis patients. Mult Scler.

[50] Khromov A, Segal M, Nissinoff J, Fast A (2005). Migraines linked to interferon-beta treatment of multiple sclerosis. Am J Phys Med Rehabil.

[51] Fragoso YD, Adoni T, Gomes S, Goncalves MV, Matta AP, Mendes MF (2015). Persistent headache in patients with multiple sclerosis starting treatment with fingolimod. Headache.

[52] Pollmann W, Erasmus LP, Feneberg W, Straube A (2006). The effect of glatiramer acetate treatment on pre-existing headaches in patients with MS. Neurology.

